# First case report of bacteremia caused by *Solobacterium moorei* in China, and literature review

**DOI:** 10.1186/s12879-019-4359-7

**Published:** 2019-08-20

**Authors:** Wen-Jing Liu, Meng Xiao, Jie Yi, Ying Li, Timothy Kudinha, Ying-Chun Xu

**Affiliations:** 1 Department of Clinical Laboratory, Peking Union Medical College Hospital, Chinese Academy of Medical Sciences, No.1 Shuaifuyuan, Dongcheng District, Beijing, 100730 China; 2 Beijing Key Laboratory for Mechanisms Research and Precision Diagnosis of Invasive Fungal Diseases, Beijing, China; 3 Charles Sturt University, Leeds Parade, Orange, Sydney, NSW Australia; 4 Centre for Infectious Diseases and Microbiology LaboratoryServices, ICPMR-Pathology West, Westmead Hospital, Westmead, NSW Australia

**Keywords:** *Solobacterium moorei*, Blood stream infection, China

## Abstract

**Background:**

*Solobacterium moorei*, the only species in the genus *Solobacterium*, is a Gram-positive, non-spore-forming, strict anaerobic, short to long bacillus. It has rarely been documented to cause blood stream infections. Here we report the first case of bacteremia caused by *S.moorei* in China.

**Case presentation:**

A 61-year-old male presented to Peking Union Medical College Hospital (Beijing) with thrombotic thrombocytopenic purpura (TTP) and several other underlying diseases. He also had persistent coma accompanied by intermittent convulsions, halitosis, and intermittent fever. Blood cultures taken when the patient had a high fever were positive, with the anaerobic bottle yielding an organism identified as *S.moorei* by 16S rRNA gene sequencing, whilst the aerobic bottle grew *Streptococcus mitis*. After replacement of venous pipeline, and empirical use of vancomycin and meropenem, the patient’s body temperature and white blood cell count returned to normal. Unfortunately, the patient died of severe TTP.

**Conclusion:**

This is the first case report of *S. moorei* isolation from blood stream in China. 16S rRNA gene sequencing is the only method that can identify *S. moorei*. Blood cultures must be taken before administration of antibiotics, and anaerobic culture should be considered for such rare pathogens in patients with oral diseases and immune deficiency.

## Background

*Solobacterium moorei* (*S. moorei*) is a member of the indigenous human intestinal microflora, and was first isolated from human feces in 2000 [1]. The new genus *Solobacterium*, which belonged to the *Clostridium* cluster XVI, was created after 16S rRNA gene sequence analysis [2]. *S.moorei* is the only species in the genus, and shows close phylogenetic relationship with *Bulleidia extructa*, *Holdemania filiformis*, and *Erysipelothrix rhusiopathiae* [1, 2]. *S.moorei* has been reported to cause halitosis [3–5], other oral cavity diseases [6–9] and wound infections [10]. To our knowledge, there are only 4 reports on blood stream infection caused by *S.moorei* [11–14]. Herein, we present the first case of *S. moorei* bacteremia in China.

## Case presentation

A 61-year-old man who previously worked in a cattle farm, and had a medical history of hypertension for more than 10 years, hyperlipidemia, type 2 diabetes, rectal cancer treated with 6 rounds of chemotherapy, and brucellosis, presented to a local primary hospital. On April 12,017, he had a fever (38.5 °C), cough, expectoration, dizziness, headache and fatigue. Pneumonia was suspected by Computed Tomography (CT) test at the local primary hospital. The symptoms did not improve after cefuroxime and moxifloxacin treatment for 3 days. On April 42,017, the patient developed seizures and unconsciousness, and was then transferred and admitted to the emergency department of Peking Union Medical College Hospital (PUMCH). Laboratory tests revealed that the patient’s white blood cell count was 5.67 × 10^9^/L with 63% neutrophils, hemoglobin was 11.91 g/L, platelet count was 16.0 × 10^9^/L, and procalcitonin (PCT) was < 0.5 ng/mL. The patient was treated with moxifloxacin for infection, and midazolam for sedation and recurrence of convulsions. He was also transfused with platelets and put on a ventilator.

On April 52,017, further lab examinations found that brucellosis Rose Bengal Test was positive, and thus minocycline and rifampicin administration were initiated for brucellosis treatment. The patient was also diagnosed with thrombotic thrombocytopenic purpura (TTP) syndrome with a very low platelet count of 16.0 × 10^9^/L for which methylprednisolone and continuous plasma exchange were commenced to treat the patient. On April 132,017, sputum culture yielded extended-spectrum β-lactamase (ESBL) positive *Klebsiella pneumoniae* and ESBL negative *Proteus mirabilis*. Microbiology tests from other specimens (cerebrospinal fluid, bone marrow, femoral vein catheter blood and jugular vein catheter blood) were all negative from April 5 to April 132,017. The patient had serious halitosis as reported by the doctor in charge, but no pathogen was isolated from the oral secretion. On April 5 and 7, three sets of blood cultures were taken but all were negative for pathogens. On April 13, the patient developed a high fever, and another set of blood cultures was taken, and was positive after incubation for 25 h (aerobic) and 51 h (anaerobic) in the BacT/Alert automated blood culturing system. Further tests identified the cultured organisms as *Streptococcus mitis* (aerobic bottle) and *S.moorei* (anaerobic bottle). Treatment was adjusted accordingly, which included replacement of venous pipeline and empirical use of vancomycin and meropenem. The patient’s body temperature and white blood cell count returned to normal levels. Unfortunately, the patient had been in a coma since admission, and on April 21, the patient died of severe TTP.

## Microbiology and molecular examination

*S.moorei* is a strict anaerobic organism which can only grow under anaerobic conditions. On blood agar it forms gray white, non-hemolytic colonies (around 0.5 mm in diameter) after 72 h of incubation at 37 °C. Gram staining morphology revealed short to long non-spore-forming Gram-positive bacilli. *S.moorei* grows slowly, produces relatively few positive biochemical reactions, and phenotypic variations appear to be commonly exhibited by different strains. Due to these challenges, *S.moorei* cannot be identified using any commercially available identification kits [15]. Moreover, the organism cannot be identified by Vitek (bioMérieux) or Bruker Matrix-Assisted Laser Desorption/ Ionization Time-of-Flight (MALDI-TOF) as the organism’s spectrum is unavailable in the respective databases.

Antimicrobial susceptibility of *S.moorei* was determined by the E-test gradient method according to the manufacturer’s instructions. Brucella blood agar supplemented with hemin and vitamin K was used as the primary plate, and *Bacteroides fragilis* ATCC 25285 was used as the quality control strain. The breakpoints for antimicrobial agents tested were according to Clinical and Laboratory Standards Institute (CLSI) guidelines 27rd informational supplement (M100-S27), Antimicrobial susceptibility results of *S. moorei* are shown in Table 1.

**Table 1:** Antimicrobial susceptibility results of *S. moorei*

Antimicrobial agent	MIC (μg/ml)	MIC breakpoints	Interpretive Categories
Penicillin	0.003	*S* ≤ 0.5 I = 1 *R* ≥ 2	S
Meropenem	0.032	*S* ≤ 4 I = 8 *R* ≥ 16	S
Vancomycin	0.25	–	S*
linezolid	12	–	–

“-”: There is no breakpoint for Vancomycin according to the CLSI

*: Interpretive Categories was according to Pedersen RM et al. [14]

**Fig. 1:**
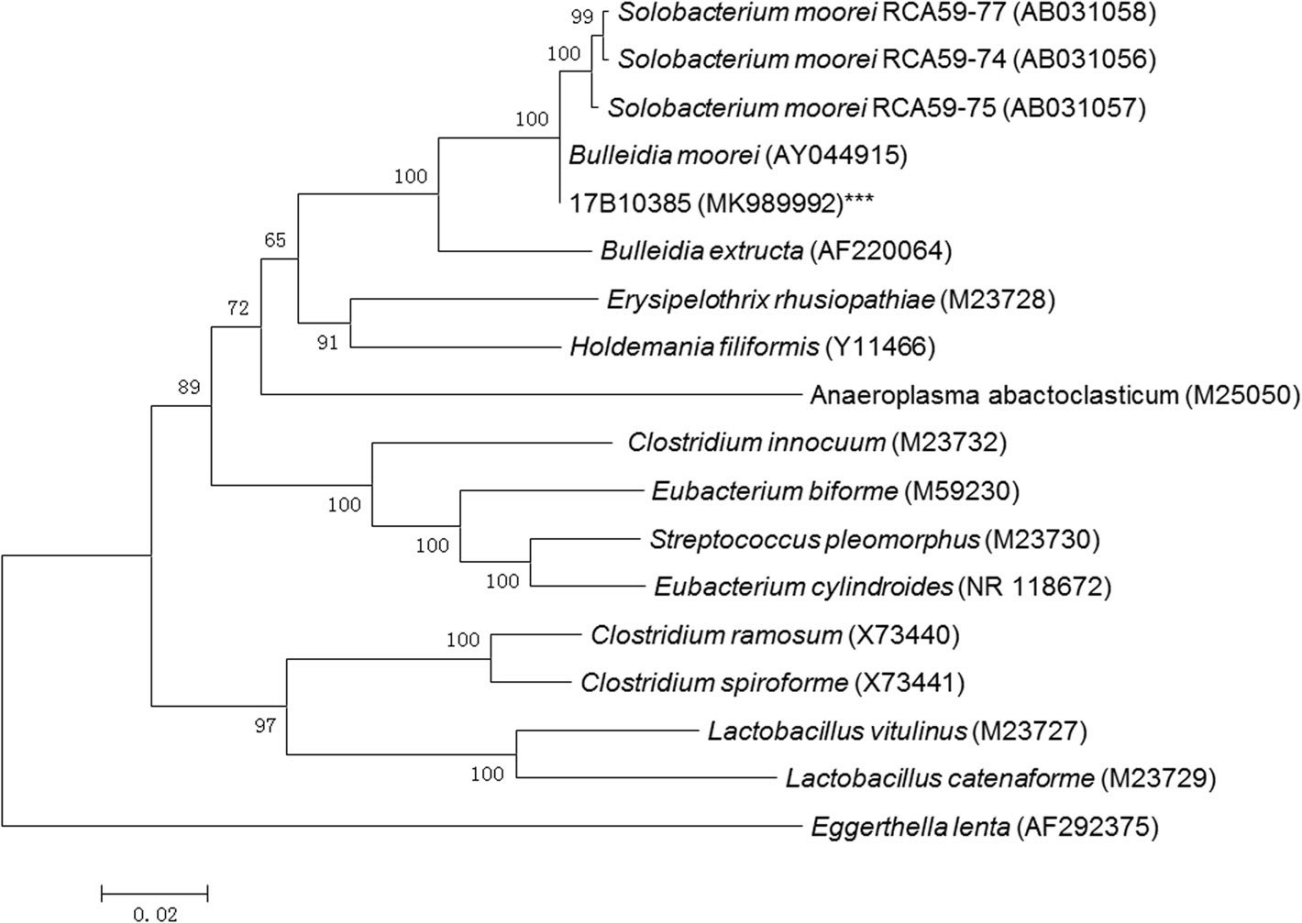
Phylogenetic tree showing the relationships of the blood culture isolate to *S. moorei* isolates and members of other related genera. The tree was constructed by using the neighbour-joining method and bootstrap values calculated from 1000 trees. The accession numbers shown are those in the GenBank database. ***: the clinical *S.moorei* isolate 17B10385 in this study

16S rRNA gene sequencing was performed to reliably identify the organism. The universal primers were 27F (5′-AGAGTTTGATCCTGGCTCAG-3′) and 1492R (5′- TACGGCTACCTTGTTACGACTT − 3′), generating a sequence with 1403 base pairs. BLAST analysis matched the strain to *S.moorei* strain JCM 10645 with identity similarity of 99.9%. We also constructed a phylogenetic tree [16] of the clinical isolate 17B10385 in this study with *S.moorei* from GenBank and closely related genera [1, 12]. The phylogenetic tree analysis also confirmed our clinical isolate as *S.moorei* (Fig. 1). The 16S rRNA gene sequence of our *S.moorei* strain has been deposited in GenBank (accession number MK989992).

## Discussion and conclusions

Early studies showed that *S.moorei* is phylogenetically related to *Eubacterium. Eubacterium* includes all anaerobic, non-spore-forming, Gram-positive bacilli except *Propionibacterium*, *Lactobacillus* and *Bifidobacterium*. The main glucose fermentation product for *S.moorei* is acetic acid, whilst for *Propionibacterium*, *Lactobacillus*, *Bifidobacterium* and *Eubacterium*, it is propionic acid, lactic acid, acetic acid, both butyric acids and formic acid [1]. Due to differences in culture conditions, G + C contents and fermentation products, *S.moorei* was assigned to a new species.

In the last decade, *S. moorei* has mainly been reported to cause oral diseases, including halitosis [3–5], endodontic infections [6, 7], peri-radicular lesions [8], refractory periodontitis [9], root canals [7], periodontal disease and dentoalveolar abscesses [2]. Studies show that *S.moorei* mainly colonizes the oral cavity of halitosis patients, with detection frequency in healthy volunteers much less than in halitosis patients. The distribution of *S.moorei* varies by race and gender, being more prevalent in African Americans, followed by Hispanics and Whites, and in males than females [3–5]. *S.moorei* adheres to oral epithelial cells through adhesins. Biofilm formation is a key step in the development of halitosis [17]. Furthermore, *S.moorei* produces beta galactosidase and hydrogen sulfide, which play important roles in halitosis [4].

In addition to oral diseases, *S.moorei* is reported to cause wound and blood stream infections, although this is relatively rare. To date, there are only four reports about *S.moorei* bacteremia [11–14], and one report on wound infection [10]. Table 2 summarizes *S. moorei* bacteremia and wound infections. Including the present case, there are only 9 cases of *S. moorei* bacteremia; 5 cases of *S. moorei* bacteremia were reported by Pedersen during a period of 7 years [14]. *S. moorei* deposited in the clone library was isolated from only 9 cases in a pool of 400 surgical wound infections. Clinical data indicates that patients with compromised immunity (such as malignant disease, intravenous drug abuser, diabetes and history of surgery) are more susceptible to *S.moorei* bacteremia across gender and age. *S.moorei* is susceptible to commonly used anti-anaerobic agents (e.g., penicillin, piperacillin-tazobactam, clindamycin, metronidazole, meropenem, moxifloxacin, tegacycline and vancomycin). For wound infections, surgical debridement and drainage is commonly required. In the present case, the high fever developed during treatment may be related to the entry of *S.moorei* and *Streptococcus mitis* into the bloodstream. After replacement of venous pipeline and empirical use of vancomycin and meropenem, the patient’s body temperature and the total leukocyte count returned back to normal.

**Table 2:** Review of published *S. moorei* literature^a^and one case from our hospital over the past 12 years

Case no.	Age (y),Sex	Symptoms	Underlying disease	Complication/Focus	Treatment	Surgery	Outcome	Reference no.
bacteremia1	67, M	unconscious, feverish, unstable hemodynamic state, severe hypotension and atrial fibrillation	multiple myeloma	Not reported	cefepime	no	Recovered	11
bacteremia2	43, F	fever, chills, and rigor associated with vomiting, lower abdominal and anal pain, and watery diarrhea	Acute Proctitis and Carcinoma of the Cervix	Not reported	Piperacillin-tazobactam	no	Recovered	12
bacteremia3	37, M	pain in his right groin, fever, rigors and vomiting	intravenous drug use	Femoral vein thrombophlebitis and septic pulmonary embolism	penicillin and metronidazole	no	Recovered	13
bacteremia4	43, M	fever, anemia, diarrhea, and general malaise and was complainingabout a toothache.	lymphoma and a kidney transplantation	Not reported	benzylpenicillin and metronidazole	no	Recovered	14
bacteremia5	66, F	fever and fatigue	non-small-cell lung carcinoma	meningeal carcinomatosis, septic with low blood pressure, pulmonary abscess	cefuroximeand gentamicin firstly, and then changed to meropenem and metronidazole, ciprofloxacin and metronidazole finally	no	Not reported	14
bacteremia6	64, M	fever and signs of gastrointestinal atony	colon cancer and complicated abdominal surgery	septic with low blood pressure	cefuroxime and metronidazole	no	discharged from hospital	14
bacteremia7	33, F	fever, headache, and skin numbness	intravenous drug abuse and hepatitis B	thrombosis of the left femoral vein and an abscess	cefuroxime firstly, and then changed to benzylpenicillin and metronidazole	no	Recovered	14
bacteremia8	77, M	fever, dry cough, and general discomfort and had been complaining about a toothache	ischemic heart disease and cancer of the prostate	Pneumonia, hypotension	benzylpenicillin	no	Recovered	14
bacteremia9	61, M	fever, cough, expectoration, dizziness, headache and fatigue, serious halitosis	TTP, hypertension, hyperlipidemia, type 2 diabetes, rectal cancer and brucellosis	Pneumonia, persistentcoma, accompanied by intermittent convulsions	vancomycin and meropenem	no	discharged from hospital	present case
wound infection	Not reported	nine cases of surgical wound infection with mixtures of aerobic and anaerobic bacteria involving *S. moorei*	Perforated appendix, ventral hernia, diabetes mellitus, intravenous drug use	Left thigh Spontaneous abscess of 3 wks’ duration, Abdominal wound abscess, right axilla Furuncle, Abdominal wound infection, Perirectal abscess, Infected pilonidal cyst, Right thigh abscess, Pilonidal abscess	various antimicrobial regimens	yes	Recovered	10

^a^Summary of the nine cases of *Solobacterium moorei* bacteremia and nine cases of wound infection reported in literature

*S.moorei* bloodstream infection may originate from oral infection, lung abscess, abdominal infection, and the habit of licking needle by intravenous drug users. The patient in this case had serious halitosis, which maybe the origin of the bacteremia. However, we failed to culture this pathogen from oral secretions, possibly due to the fact that oral secretions are not routinely cultured for anaerobic pathogens. Thus for patients with oral diseases such as halitosis, anaerobic cultures should be strongly recommended to identify suspicious pathogens.

The newly discovered *S. moorei* was isolated from patients with oral diseases and immune deficiency. Although rarely isolated, its pathogenicity in oral, wound and bloodstream infections is very clear. *S. moorei* exhibits susceptibility to common antibiotics used for anaerobic infections. Prior use of antibiotics before blood cultures are taken may yield negative results. Due to the special culture conditions and specialized identification method, the prevalence of *S. moorei* bacteremia may be underestimated. Laboratory staff and clinicians should pay more attention to such rare bacteria and their clinical significance. Optimization of blood culture procedures and utilization of 16S rRNA gene sequencing are powerful tools for rare pathogen identification from blood and other sterile body fluids.

## Data Availability

The datasets used and/or analyzed during the current study are available from the corresponding author on reasonable request.

## Abbreviations

CLSI: Clinical and Laboratory Standards Institute
CT: Computed Tomography;
ESBL: Extended-Spectrum β-Lactamases
MALDI-TOF: Matrix-Assisted Laser Desorption/Ionization Time-of-Flight
MIC: Minimal Inhibitory Concentration.
PCT: Procalcitonin;
TTP: Thrombotic thrombocytopenic purpura
